# *Notes from the Field:* Cluster of Parechovirus Central Nervous System Infections in Young Infants — Tennessee, 2022

**DOI:** 10.15585/mmwr.mm7130a5

**Published:** 2022-07-29

**Authors:** Lili Tao, Mary-Margaret A. Fill, Ritu Banerjee, Romney M. Humphries

**Affiliations:** ^1^Department of Pathology, Microbiology and Immunology, Vanderbilt University Medical Center, Nashville, Tennessee; ^2^Communicable and Environmental Diseases and Emergency Preparedness Division, Tennessee Department of Health; ^3^Division of Pediatric Infectious Diseases, Department of Pediatrics, Vanderbilt University Medical Center, Nashville, Tennessee.

During April 12–May 24, 2022, 23 previously healthy infants aged 5 days–3 months were admitted to a Tennessee children’s hospital for human parechovirus (PeV) meningoencephalitis.* PeV is a nonenveloped RNA virus of the Picornaviridae family. PeV infections range from mild, self-limiting gastroenteritis to severe sepsis-like disease and central nervous system (CNS) infection, (*1*) and infants aged <3 months are disproportionately affected. PeV genotype 3 is responsible for the most severe cases, with a pattern of biannual cycle circulation that peaks during summer months (*2*,*3*). Although PeV infection is not a reportable disease, the Tennessee Department of Health was notified. An assessment of cases was conducted to better understand this unusually large cluster of infections.

At this children’s hospital, a lumbar puncture is performed as part of sepsis evaluation for all infants aged <1 month and for older infants when clinically indicated. Cerebrospinal fluid (CSF) testing includes a multiplex molecular panel (BioFire FilmArray Meningitis/Encephalitis Panel, bioMerieux) for all infants aged ≤3 months and for patients aged >3 months if the CSF white blood cell (WBC) count is >5 cells per high power field. For this investigation, a comprehensive review of electronic health records was conducted to assess demographic characteristics, social history, signs and symptoms at admission, laboratory test results, and treatment course of all patients in whom PeV was detected by the multiplex molecular panel during the cluster period. This study was reviewed and approved by the Vanderbilt University Medical Center Institutional Review Board and was conducted consistent with applicable federal law and CDC policy.^†^

Median age of the patients was 24 days; 13 (57%) were female and 10 (43%) were male (Table). Five patients were preterm (28–36 weeks’ gestation). Signs and symptoms included fever, fussiness, and poor feeding. Most patients became symptomatic in the community (22, 96%); one preterm infant became symptomatic while in the neonatal intensive care unit (NICU). One (4%) patient attended a child care facility, and 16 (70%) had siblings at home or were exposed to other children. 

**TABLE:** Characteristics of infants with parechovirus central nervous system infection (N = 23) — Nashville, Tennessee, April 12–May 24, 2022

Characteristic	No. (%)
**Sex**	
Female	13 (57)
Male	10 (43)
**Median age (range), days**	24 (5–99)
**Gestational age at delivery**	
Preterm (28–36 wks)	5 (22)
Term (37–40 wks)	18 (78)
**Acquisition of infection**	
Community	22 (96)
NICU	1 (4)
**Exposure to other children**	16 (70)
**Signs and symptoms**	
Fever	20 (87)
Fussiness	13 (57)
Poor feeding	8 (35)
Sleepiness	4 (17)
Respiratory distress	4 (17)
Rhinorrhea, congestion	3 (13)
Seizure	1 (4)
Rash	1 (4)
Elevated CSF WBC count*	7^†^ (32)

**Abbreviations**: CSF = cerebrospinal fluid; NICU = neonatal intensive care unit; WBC = white blood cell.

* CSF cell count performed for 22 of 23 patients.

^†^ Contamination during collection was presumed for three specimens.

Leukopenia was detected in only four (17%) patients. CSF cell count was performed for 22 patients; seven (32%) specimens demonstrated an elevated WBC count, including three with probable blood contamination during collection. All but one of the infants were admitted to the hospital; four (17%) infants developed severe disease that required treatment in the NICU. Brain magnetic resonance imaging was performed in four severely ill NICU patients, which detected diffusion within the white matter consistent with typical PeV meningoencephalitis in all of these patients. Antibiotics were initially prescribed for the 23 patients but were discontinued for 13 (57%) within 24 hours of detection of PeV. The mean hospital stay was 4.5 days (range = 1–26 days). Twenty-one (91.3%) patients recovered without complications. One patient was scheduled for a 6-month follow-up for possible late onset hearing loss and hypercoagulation evaluation. One patient experienced persistent seizures and was anticipated to experience severe developmental delay.

The multiple molecular panel had been introduced at the children’s hospital in May 2018 to aid in the diagnosis of potential pathogens among patients with suspected meningitis or encephalitis. Nineteen cases were detected over 5 months in 2018, likely representing a baseline incidence of PeV CNS infections. Seven cases of PeV were detected during 2019–2021. The absence of a biennial peak in 2020 is presumably because of social isolation during the COVID-19 pandemic, suggesting that PeV transmission is closely associated with social activity. However, 29 cases were detected in 2022 at the children’s hospital, including the 23 cases described in this report that were detected within a 6-week period. This peak in infections might reflect relaxation of COVID-19 isolation measures, consistent with increased prevalence of other respiratory viruses (e.g., respiratory syncytial virus)^§^ (*4*,*5*). When PeV is circulating, clinicians should consider testing for PeV in young infants, including those with normal CSF parameters.^¶^ The rapid detection of PeV in CSF by multiplex molecular panels can limit antibiotic administration and improve patient management. Parents with young infants, especially those with infants aged <3 months, should be aware of the symptoms and visit a pediatrician if symptoms persist. 

## Footnotes

* Infants were hospitalized at the Monroe Carell Jr. Children’s Hospital, Vanderbilt University Medical Center, Nashville, Tennessee.

† 45 C.F.R. part 46, 21 C.F.R. part 56; 42 U.S.C. Sect. 241(d); 5 U.S.C. Sect. 552a; 44 U.S.C. Sect. 3501 et seq.

§
https://www.cdc.gov/rsv/index.html

¶
https://emergency.cdc.gov/han/2022/han00469.asp
